# Using an integrative approach to investigate the evolution of behaviour

**DOI:** 10.1111/eva.12300

**Published:** 2015-09-21

**Authors:** Nadia Aubin‐Horth

**Affiliations:** ^1^ Département de biologie & Institut de Biologie Intégrative et des SystèmesUniversité LavalQuébecQCCanada

**Keywords:** adaptation, behaviour, integrative biology, phenotypic plasticity, transcriptomics

## Abstract

Behaviour is a central focus of interest in biology because it has an impact on several aspects of an organism's life. Evolutionary biologists have realised the advantage of an integrative approach that jointly studies the molecular, cellular and physiological levels of an individual to link them with the organismal behavioural phenotype. First, this mechanistic information helps in understanding physiological and evolutionary constraints acting on the behavioural response to the environment and its evolution. Second, it furthers our understanding of the process of molecular convergent evolution. Finally, we learn about natural variation in molecular, cellular and physiological traits present in wild populations and their underlying genetic basis, which can be a substrate for selection to act on. I illustrate these points using our work on behaviour variation in fishes. The information on the mechanistic bases of behaviour variation in various species and behaviours will contribute to an ecological annotation of genes and to uncover new mechanisms implicated in how this astonishing behavioural diversity arose, is maintained and will evolve.

## Integrative biology to understand behaviour diversity

Behaviour is a central focus of interest in ecology and evolution because it has an impact on several aspects of an organism's life that affects its fitness. In this context, one of the main goals of biology is to understand the evolutionary and mechanistic causes of behaviour diversity, which is found among life stages, individuals, populations and species (Tinbergen 1963). Evolutionary biologists have traditionally focused on uncovering the ultimate causes that lead to the evolution of behaviour (natural selection, genetic drift and phylogenetic constraints). Evolutionary biologists have also concentrated on environmental effects on the development of behaviour (see Box 2) and the proximal triggers that affect behaviour, including challenges and opportunities faced by an individual (competition, predation, resource availability and potential mate, O'Connell and Hofmann 2011). Originally in mostly separate efforts, biologists have asked fundamental questions about the mechanistic basis of behaviour (see Box 3 for details). This includes understanding the neurobiology of behaviour and the function of molecules in a given organism and environmental context (*sensu* ‘selected effects’, Doolittle et al. 2014) and how they may modulate the response to internal and external cues integrated by the nervous system. This response then governs the neural mechanisms that control the behavioural output we observe (Soares et al. 2010).

An integrative biology approach emerges from the fusion of these evolutionary and mechanistic questions. Interests of biologists studying the evolution of behaviour and of the ones studying its mechanistic basis are converging, as we realize that these processes are tightly linked and that understanding one aspect better will help shedding light on the other. The particularity of an integrative approach is to jointly study the molecular, cellular and physiological levels of an individual to link it with the organismic phenotypic variation of interest, to answer fundamental questions in ecology and evolution (Wake 2003; see Box 4). One of the interesting aspects of focusing on these molecular, cellular and physiological mechanisms is that they can be studied for behaviour variation that results from genetic variation and from phenotypic plasticity (Aubin‐Horth and Renn 2009; Dalziel et al. 2009; Box 2). Using a hierarchical approach by studying these different levels together while simultaneously keeping the focus centred on the organism allows us to not only understand how they work together and how they influence each other, but also to uncover the emerging properties of each of these levels (Novikoff 1945; Wake 2003).

In this review, I would like to illustrate how studying the mechanistic basis of behaviour variation provides gains on multiple fronts for evolutionary biologists. I will use examples from my own research programme to show how using an integrative biology approach, by combining training in organismic evolutionary biology, behavioural biology, functional genomics and physiology, has helped me uncover new knowledge about the evolution of behaviour. First, studying the mechanistic bases of behaviour variation helps in understanding physiological and evolutionary constraints acting on the behavioural response to the environment and its evolution (Stearns and Magwene 2003; Sih et al. 2004). Specifically, I will illustrate this using the trade‐offs seen in life histories in a species with alternative life cycles, the Atlantic salmon. Second, studying the mechanistic bases of behaviour variation provides information on the process of molecular convergent evolution. It allows us to test whether the same molecular signalling networks are reused in different species to give the same phenotype (Starnecker and Hazel 1999; Arendt and Reznick 2008; Stern 2013). I will illustrate this goal using studies of social behaviour in African cichlids. Finally, understanding the evolution of behaviour necessitates understanding the natural variation available for selection to act on. This natural variation is underlined by variation in molecular mechanisms, which we know surprisingly little about. I will use examples from our studies of natural variation in threespine sticklebacks to illustrate the additional information gained about behaviour when the organismic and molecular levels are considered simultaneously. Finally, these examples from my career will also serve to present the emergence of a new research field in the last 20 years, ecological genomics (Landry and Aubin‐Horth 2014; see Box 1).

Box 1 **Personal reflections**

The editors of this special issue asked us to present our thoughts on being a woman in evolutionary biology and in science in general. First, I must say that my background affects my view on life as a woman in science. I am a North American (French speaking) white woman, raised by two parents with university faculty positions and high socioeconomic status, growing up with a little sister that now has a PhD. I was strongly encouraged by them to pursue a scientific career, and they supported me morally and financially all the way through my undergraduate and graduate studies. The influence of my privileged background on my chances of success never escapes me, and I try every day to make the most of the opportunity I was offered and to recognize when other scientists that cross my path face a different reality. However, as a woman in science, I still face a disadvantage: recent data show that woman scientists receive smaller grants on average than their male counterparts in the same competitions, are nominated less often for prestigious awards and are in minority on editorial boards (Cho et al. 2014) and in high‐ranking positions. In Canada, ‘women have been awarded only 17% of major Natural Sciences and Engineering Research Council of Canada (NSERC) awards since 2004’ (for detailed numbers and explanations see Bond 2014). For example, according to a NSERC report on Women in Science and Engineering in Canada, between 2000 and 2010, the Steacie prize for Natural Sciences, which is offered to a promising young scientist in Canada, has been awarded 73% of the time to men (NSERC 2010)! Canadian woman scientists also receive lower NSERC Discovery grants on average, as presented by Prof. Judy Myers based on NSERC data (Myers 2014). Fortunately, funding agencies such as NSERC, universities and scientists are aware of these important hurdles and are looking for solutions. Based on my own career and experience, one solution would be to redefine how we measure success and the perceived traditional ways of achieving it, a question that has been discussed recently at The Symposium for Women Entering Ecology and Evolution Today (SWEEET, see http://sweeetecoevo.weebly.com/), organized annually in parallel with the Canadian Society for Ecology and Evolution meeting. Such a shift in thinking would be advantageous for scientists of all genders and backgrounds who may have career paths that do not comply with the obsolete, established norm, but that have much to offer to the world of science and in general. Increasing diversity and equity is a goal that will benefit everyone.

This text stems from an invitation to write for a special issue on ‘Women's contribution to basic and applied evolutionary biology’. I have chosen to present my professional career trajectory in ecological genomics as an illustration, in combination to presenting a research theme I find fascinating. I therefore mostly cite only my own work and discuss the various steps in my career as an evolutionary biologist. Writing about oneself is probably the hardest and most awkward thing I have done in my career and I hope the reader will appreciate the special context in which I wrote this review.

## An organismal evolutionary biologist' s journey into the realm of the invisible

### 1‐Alternative life cycles in Atlantic salmon

#### Evolution of alternative reproductive tactics

My PhD in Biology (1997–2002) in the laboratory of Julian Dodson in the Biology Department at Université Laval focused on the evolution of an alternative reproductive tactic in male Atlantic salmon (Taborsky et al. 2008). This species is anadromous. Its life cycle is split between freshwater and saltwater. Reproduction takes place in freshwater; juveniles spend months to years in this habitat and then go through major physiological, morphological and behavioural changes while transitioning to the saltwater environment. After a period of fast growth in saltwater, adults migrate back most frequently to their river of origin and reproduce (Webb et al. 2007). However, we now know that two types of reproductive males are actually present in this species: large fighter males and small sneaker males (see Dodson et al. 2013 for a complete review in salmonids). These two alternative reproductive tactics both allow access to reproduction but entail different life‐history trade‐offs (Stearns 1992). Large males perform a migration to saltwater before reproduction. Large males fight among themselves for access to female, and the dominant fighter male has a high fertilization success (Webb et al. 2007). The alternative sneaker male type does not migrate to saltwater before reproducing. These males are the size of juveniles but divert energy from somatic growth and lipid storage towards gonad development (Saunders et al. 1982; Moore and Scott 1991 Hutchings and Myers 1994; Waring et al. 1996). They do not fight for access to females but rather sneak into the nest built by females to fertilize eggs while hidden from the large males and females. These sneaker males have a much lower fertilization success than the dominant males but their survival to reproduction is higher (Webb et al. 2007).

These alternative male reproductive tactics are the result of phenotypic plasticity (Hutchings and Myers 1994; West‐Eberhard 2003). My PhD project focused on determining whether the genotype and the environment affect early life size and whether size predicts which life history a male will develop (Aubin‐Horth and Dodson 2004; Dodson et al. 2013). Size attained during a specific developmental window in the spring preceding reproduction in the fall allows us to predict whether a male will mature sexually as a sneaker (Whalen and Parrish 1999). Size is thus a good approximation of an unknown underlying developmental switch mechanism (Dodson et al. 2013). Males below a certain threshold condition will allocate energy to growth and energy reserves. Males above the threshold condition will allocate energy to gonad development and will develop as sneakers (Hazel et al. 1990; Hutchings and Myers 1994; Aubin‐Horth and Dodson 2004; Garcia de Leaniz et al. 2007). Sneaker males are thus the largest in the spring, then divert energy from growth to gonad development, resulting in a lower subsequent growth than the males that stayed immature (Whalen and Parrish 1999; Arndt 2000; Aubin‐Horth and Dodson 2004). Interestingly, we showed that subpopulations in large river systems vary in the ‘threshold’ size at which an individual will develop as a sneaker male instead of continuing growth as an immature male (Aubin‐Horth and Dodson 2004). Individuals in rearing sites further upstream in the river (tens of kilometres) have a lower threshold size and thus more males reach the threshold and become sneakers for the same average size (Aubin‐Horth et al. 2006). Furthermore, we uncovered that size‐selective mortality can affect the average size in a cohort and change the proportion of males developing as sneakers (Aubin‐Horth et al. 2005c). A condition threshold also determines the development of the migratory phenotype in the spring, such that individuals migrate at the same size but not necessarily the same age (Reviewed in Dodson et al. 2013). However, sneaker males have a significantly lower probability of migrating to saltwater in the spring that follows the fall reproductive period compared to individuals of equal size that did not mature the previous fall (Whalen and Parrish 1999; Letcher et al. 2002; Dodson et al. 2013). The lower probability of migration for sneakers compared to immature individuals of the same size suggests a physiological trade‐off between these two life‐history stages. These trade‐offs and their evolution have been the central topic of the study of life‐history evolution (Stearns 1992), but the underlying mechanisms leading to these trade‐offs, for example conflicts in hormonal levels and gene expression, or pleiotropy, had not been studied at the time and had to be treated as ‘black boxes’ (Stearns and Magwene 2003).

#### Opening the black box

During my training in the PhD Biology programme, I learned that the alternative reproductive tactics in salmon were an excellent model to study evolution of life histories. However, it had also led me to bump into the ‘black box’ (Stearns and Magwene 2003) of how these fascinating phenotypes develop. I thus had more questions than answers when I was finishing my degree. It was clear to me that we needed to understand how individuals with very similar genotypes would develop into such divergent phenotypes, if we aspired to understand the evolution of this behavioural plasticity. I was very interested in understanding how the brain of a sneaker male would change during development compared to immature males. The brain was the most interesting tissue to focus on to study gene expression because it acts as a hub. It integrates external and internal stimuli and controls outgoing signals that result in the phenotype at the molecular, cellular, physiological, morphological and behavioural level. I also wanted to understand how the apparent trade‐off between maturing as a sneaker male in the fall and the probability of saltwater migration the following spring was translated at the molecular level. Could we uncover information about this striking trade‐offs in energy partitioning by studying gene expression (as done in *Drosophila*, Bochdanovits and de Jong 2004)? Could we open the ‘black box’ and measure conflicts in gene expression, as proposed by Stearns and Magwene (2003)? All these questions led me to seek training in a very different field, functional genomics, as a postdoctoral fellow at the Bauer Center for Genomics research at Harvard University in the laboratory of Hans Hofmann.

#### The beginning of ecological genomics

When I started my postdoctoral fellowship (2002), the first microarray usable to study the expression of thousands of genes in a single experiment had been described in the previous years (Schena et al. 1995). It was thus possible to quantify the association between variation in a phenotype and in gene expression at a genomewide scale (Drosophila, Jin et al. 2001; fish, Oleksiak et al. 2002). The first draft of the human genome had just become available (International Human Genome Sequencing Consortium 2001). Life science researchers were ready to take advantage of the array of high‐throughput biomedical molecular tools to answer question in their field (Gibson 2002; Stearns and Magwene 2003). In the case of alternative life cycles in salmon, we wanted to study molecular changes associated with a plastic trait. It was thus key to quantify the mechanisms at biological levels that are labile within the life of an individual, such as mRNA levels (Aubin‐Horth and Renn 2009). The high‐throughput molecular tools, such as microarrays, allowed evolutionary biologists to test new hypotheses by studying the expression of hundreds of candidate genes simultaneously. This large‐scale view gave new information on their variation in expression across phenotypes, as well as on their covariation in expression with each other. By taking advantage of having information for thousands of genes at a time, we could also test hypotheses as to which and how biological processes should respond in coordination with each phenotype, based on life‐history evolution theory. Finally, we could explore and uncover new candidate genes and biological processes associated with these phenotypes of interest, in a range of model species in ecology and evolution. The field of ecological genomics was born (Nevo 2001; Gibson 2002; Feder and Mitchell‐Olds 2003), and I was there to see it hatch out of its shell. Of course, fifteen years later, we now use different molecular tools than what was available in the early 2000s. It is indeed easy to predict that a high‐throughput technology that seems cutting edge now will certainly be replaced by newer, better methodologies, but fortunately the principles of what we can learn about the evolution of behaviour by studying mechanisms is not technology centric.

#### Ecological genomics of trade‐offs in alternative life cycles

We studied two dramatic life‐cycle transitions in Atlantic salmon. We were interested in the associated energy allocation trade‐offs measured at the organismic level and predicted by life‐history evolution theory (Stearns 1992). We first focused on the life stage at which juveniles develop as sneaker males or stay immature, to study the growth – reproduction trade‐off. We also studied the life stage at which individuals migrate from freshwater to saltwater or stay as resident in freshwater for an additional year. This transition is accompanied by large changes in physiology, morphology and behaviour, including changes in saltwater tolerance, hormone levels and a shift in swimming directionality (Prunet et al. 1989; Iwata 1995; Ágústsson et al. 2003; Dukes et al. 2004). Studying this life stage allowed us to uncover changes at the gene expression levels associated with these well‐known attributes related to migration. It also allowed us to investigate the apparent trade‐off between maturing in the fall and migrating to saltwater in the following spring. Studying trade‐offs at the molecular level in the context of studying life‐history evolution was just burgeoning at the time (Bochdanovits and de Jong 2004).

We studied gene expression using a custom cDNA microarray built using sequences from salmonids (Rise et al. 2004). We compared the brain transcriptome of wild males Atlantic salmon that develop as reproductive sneakers in the fall with males of the same age that stay immature (Aubin‐Horth et al. 2005a). By doing so, we characterized for the first time large‐scale gene expression in the brain of a wild vertebrate. We found that 15% of the genes surveyed varied in expression in the brain between male types. Seventeen distinct biological processes were implicated, suggesting that several molecular pathways were coregulated (Aubin‐Horth et al. 2005a). We found differences in candidate genes and biological processes expected to differ between sneaker males and immature male brains. For example, genes with functions associated with sexual development and maturity, feeding and reproduction were identified. It also revealed new processes to further study, such as the potential high cognitive demands of sneaking behaviour. This was suggested by the differential expression of genes associated with neural plasticity and neural signalling in sneaker males (Aubin‐Horth et al. 2005a). Interestingly, this surprising and unexpected result on the cognitive demands of sneaking has been recently observed in an independently evolved system of alternative reproductive behaviour in the sailfin molly (Fraser et al. 2014). This helps in formulating predictions that could be tested by direct behavioural assessments of learning and memory capacity in males of different reproductive tactics. Importantly, we supported our prediction that the trade‐off between investment in reproduction and in growth observed at the whole‐organism level would be reflected at the molecular level (Aubin‐Horth et al. 2005a). Finally, in a companion study, we compared the expression profiles of sneaker males and immature males reared in the wild and in a hatchery. This study revealed the large effects of the rearing environment on brain gene expression (Aubin‐Horth et al. 2005b).

We also studied the large‐scale transcriptome changes that occur during the transition from freshwater to saltwater. We wanted to know which changes in brain gene expression were associated with the sea migration switch point in the Atlantic salmon life cycle. There was very little information on the molecular changes happening in the brain of a migrating vertebrate, if any. We compared male and females juveniles that were predicted to migrate or stay as residents based upon morphology and coloration (Aubin‐Horth et al. 2009). In summary, genes associated with several biological functions changed in expression levels between migrants and residents, including known candidates based on previous physiology studies and new candidates, allowing us to define a brain genomic signature of migration behaviour in a vertebrate (Aubin‐Horth et al. 2009). This study also allowed us to compare the list of molecular changes in the brain found between migrants and residents with the changes found between sneaker and immature males, to determine whether the set of genes that changed in expression in these two life‐cycle stages overlapped. Interestingly, more than 70% of gene expression variation was specific to each switch point (sneaker/immature, migrant/resident), with 28 genes changing in expression in both comparisons (Aubin‐Horth et al. 2009). This result suggests that these genes that change in expression in both life stages are important in creating and maintaining vastly different phenotypes, if the association reflects a causal link. It thus also implies that the same molecules are reused in different contexts and have different effects depending on with which other molecules they are present. Within these 28 overlapping genes, genes highly expressed in the brain of mature sneaker males compared to immature males were less expressed in the brain of migrating fish compared to resident fish (Aubin‐Horth et al. 2009). This may represent a first step towards understanding the observed trade‐off between maturing as a sneaker in the fall and migrating to saltwater the following spring at the molecular level (Dodson et al. 2013).

### 2‐Social behaviour in African cichlids

#### Krogh's principle

Studying differential gene expression in the brain in a species that is not a molecular model system such as Atlantic salmon generates specific challenges. For instance, the function attributed to these genes (‘functional annotation’) is mainly based on work done in humans and other mammals. What the function of these genes is in an Atlantic salmon brain (or any other nontraditional model species) is usually inferred based on the assumption that most homologous genes have conserved functions. Therefore, in addition to describing these differences in gene expression, it is also crucial to study and uncover the functional consequences of this differential expression in Atlantic salmon. One advantage of pharmacological manipulation produced in controlled settings is to allow for hypotheses testing and to obtain a number of individuals that is high enough to provide statistical power. The availability of an annotated genome and molecular tools such as BAC libraries, genetic and physical maps is a major bonus for anyone wanting to do a follow‐up study (Pavey et al. 2012), and it was not available for Atlantic salmon. As stated by the Kroch's principle, ‘for a large number of problems there will be some animal of choice or a few such animals on which it can be most conveniently studied’ (Krogh 1929). I needed such a model. One of the classic model systems used to study the ecology and evolution of behaviour is the African cichlids (Kocher 2004). This fish family has become an ideal ecological genomics model, with a sequenced genomes and a wide array of genetic tools available (Salzburger et al. 2008; Fujimura and Kocher 2011; Guyon et al. 2012; Brawand et al. 2014). Importantly, transcriptomics tools, to study large‐scale changes in gene expression, were available as early as 2002 (Renn et al. 2004).

#### Dominance and reproduction

During my postdoctoral fellowship, we studied a species of African cichlids (*Astatotilapia burtoni)* in which males are subordinate and nonreproductive or dominant and reproductive (Renn et al. 2008). These two adult male phenotypes are the result of phenotypic plasticity and males switch between these two roles during their lifetime. The study of the changes at different levels of organization when a male *A burtoni* switches from one dominance status to another has been carefully defined through several studies (see Figure 1 in Renn et al. 2008 for a summary).

One of the interests of studying the mechanisms underlying behavioural variation is to be able to test for molecular convergence, that is whether the changes in the same molecular pathways (in mRNA amounts, protein activity, etc.) are found in several species in association with similar behaviours (Arendt and Reznick 2008). Such knowledge brings crucial information on the path taken or available for the evolution of behaviour. To reach this goal, we must first find the commonalities of behaviour that are comparable between species (Lefebvre and Sol 2008). For example, the building blocks of social behaviour, such as sociability and aggression, include simple behaviours that when combined create complex social behaviours (Soares et al. 2010). Finding these simple behaviours in different species allows to compare them. Grouping behavioural responses in terms of responses to challenge and opportunities and ‘evolutionary characters’ (O'Connell and Hofmann 2011; Araya‐Ajoy and Dingemanse 2014) are also useful. Once we agree on the similarities of behaviour, we can test whether different molecular routes are taken to build these similar behaviours in different species, or whether the same molecular network is reused over and over during evolution. Indeed, the object of selection is the phenotype, not the underlying mechanism (or genetic make‐up), so it is possible that there is more than one way to build a behaviour we see as ‘the same’. In the case of dominance in *A burtoni*, the territorial and dominance behaviour changes are striking, very well described and similar to behaviours in other vertebrate groups. However, at the time of my postdoctoral studies, the large‐scale differences in gene expression occurring in the brain between dominant and subordinate individuals were basically unexplored. We focused on brain gene expression using a custom cDNA microarray built using sequences from that species (Renn et al. 2004, 2008) to study the molecular make‐up of dominance in this species. Social behaviour and its underlying mechanisms have been studied in mammals and other vertebrates (Robinson et al. 2005; Donaldson and Young 2008). We could thus compare our findings to this wealth of information and study the presence of molecular convergence.

Many changes occur in a male that becomes dominant, confounding the effects of sexual maturation, reproductive behaviour and dominance. We separated the effects of sex and reproduction from the effect of dominance on brain gene expression by studying dominant and reproductive males, subordinate and nonreproductive males and reproductive females (who act as subordinates). We assigned genes to a ‘dominance’ and a ‘reproduction’ module. A module is defined as genes that covary in expression (Hartwell et al. 1999; Segal et al. 2004). Genes were associated with ‘dominance’ when they were more expressed in the brains of dominant males compared to subordinate males and females. Genes were associated with ‘reproduction’ when they were more expressed in dominant males and reproductive females compared to subordinate males (Renn et al. 2008). We found that several genes in the dominance module were candidates known from studies in mammals, birds and amphibians, suggesting molecular convergence in the association between differential gene expression and dominance in these vertebrates.

Previous studies uncovered an uncoupling in time of changes in behaviour, physiology, morphology and gene expression (see Figure 1 in Renn et al. 2008). Remarkably, dominant and subordinate behaviours are expressed much faster than changes at the hormonal and physiological levels (gonad maturation). Our classification of genes in the two modules helped us to understand how and whether dominance and reproductive behaviour can be expressed independently in a male. Ultimately, this information will be used to determine which constraints may act on the evolution of these behaviours. Interestingly, it is possible to manipulate females of this species to display male‐typical reproductive and dominance behaviour by removing males from the environment. We used this feature to study changes in circulating sex steroid hormones with dominance, such as androgens, independently of sex (Renn et al. 2012). This experimental system will certainly yield new insights on the genes included in the ‘reproduction’ and ‘dominance’ modules, when other levels of variation such as brain gene expression will be studied.

To define how these gene expression variations were associated with the behaviour we observed, we manipulated the molecules found to covary with behaviour to quantify whether and how this change affects behaviour. In *A. burtoni*, pharmacological manipulations allowed us to determine the interactions between different components of a system. We quantified how manipulating the action of the product of a candidate gene in the dominance module (arginine vasotocin, Renn et al. 2008) affected behaviour of dominant and subordinate males, as well as in subordinate males given an opportunity to ascend to dominance (Huffman et al. 2015). We showed that the effect of increasing or blocking vasotocin on dominance and reproductive behaviours were not symmetric and were found only in unstable hierarchies of newly ascended males. We also found that dominance and reproduction behaviours were affected differently, supporting our finding of distinct dominance and reproductive modules in our original functional genomics study (Huffman et al. 2015).

#### Cooperative breeding

In a number of mammals, birds, fish and insects, breeding pairs are assisted in rearing offspring by other individuals who live with the breeders in a social group (Solomon and French 1996). This system is known as cooperative breeding. Individuals in these social groups show distinct dominant and subordinate phenotypes. During my postdoctoral work, we studied a cooperatively breeding fish, *Neolamprologus pulcher* in collaboration with Sigal Balshine's laboratory at McMaster University. In this species, a breeding dominant pair lives on a territory where they raise their offspring in a nest. Subordinate, nonreproducing individuals help to defend the territory and the young (Taborsky and Limberger 1981; Balshine‐Earn et al. 1998). Cooperatively breeding species offer an exceptional system to study social life, notably the dominance hierarchies and social affiliation, and their effect, in an integrative manner, at the behavioural, endocrine and molecular levels. In *N. pulcher*, breeders of both sexes show high territoriality and dominance behaviour over the subordinate helper individuals. We hypothesized that quantifying what happens in the brain of dominant females, in addition to males, could help modify assumptions about the general notion of male‐specific behavioural, endocrine and molecular profiles. These assumptions are mostly due to the lack of molecular and endocrine data on females of species in which they are territorial and aggressive, as in *N. pulcher*. This model system of sociality thus gave us the opportunity to study the changes in gene expression in both males and females when they move from a subordinate helper status to a dominant breeder position (Aubin‐Horth et al. 2007). Fortunately, we had previously shown the feasibility and usefulness of using a microarray built for one species (*A. burtoni*) to study another closely related species, a method termed heterologous hybridization (Renn et al. 2004). Using this approach, we observed the largest change in gene expression in females that became dominant, as their brain expression profile most resembled the expression levels found in males (independently of the male's dominance and reproductive status). This association suggested that as they become dominant and reproductive, females express more male‐like traits, while remaining fully reproductive. This finding is in accordance with the high aggression level observed in dominant breeding females in this species (Desjardins et al. 2008) and with the hormonal changes in circulating testosterone levels also observed in dominant females (Aubin‐Horth et al. 2007). We also found that expression of the gene coding for arginine vasotocin was higher in the brain of dominant individuals of both sexes forming a pair compared to subordinate helpers. Higher expression of this gene was also found in dominant males of *A. burtoni* (Renn et al. 2008).

Taking a comparative approach will be crucial to improve our understanding of the generality of these mechanisms. To do so, we must quantify how the candidate genes uncovered in these different studies in African cichlids are associated with social behaviour in other cichlid species and in other nonmammalian vertebrates, a long‐standing question in comparative endocrinology (Barrington 1979). For example, we studied convergence in gene expression profiles of two candidate networks (the vasotocin and isotocin nonapeptides and their receptors) in four replicated instances of evolution towards sociality within the lamprologine tribe of African cichlids (O'Connor et al. 2015). Our results suggest species‐specific gene expression patterns relative to social behaviour for these candidate hormone pathways. Furthermore, high‐throughput methods have brought us a broad view that allows us to measure gene expression and proteins on a genomewide scale. This allows us to study molecular convergence not only at the level of specific genes, but also for whole molecular pathways as the object of molecular convergence (Bell and Aubin‐Horth 2010; Elmer and Meyer 2011). Defining the target of molecular convergence will be more complicated but will provide more complete portraits that reflect that molecules do not act alone and are part of complex pathways (Arendt and Reznick 2008).

#### Stable reprogramming of behaviour

*Neolamprologus pulcher* individuals must finely tune their behaviour towards each other within a tightly regulated dominance hierarchy. Social competence is strongly linked with their acceptance by their conspecifics and ultimately their fitness (Taborsky and Oliveira 2012). Altering the early social rearing environment affects several aspects of social competence in this species, and this effect persists into adulthood (Taborsky and Oliveira 2012). This effect of the early rearing environment has also been shown in birds and mammals (Bastian et al. 2003; Branchi et al. 2006, Banerjee et al. 2012). Studies have shown the effect of early life environment on epigenetic reprogramming of brain gene expression in mammals.

In 2006, I became an assistant professor in the Biological sciences department at Université de Montréal. In collaboration with the laboratory of Barbara Taborsky at the University of Bern, we studied brain gene expression to test whether there was molecular convergence in the reprogramming of gene expression in fish. We studied two groups of adults of *N. pulcher*. One group spent the first 2 months of their life in a normal social setting. The other group was raised in a socially deprived environment, that is without a dominant breeding pair but in the company of same age siblings. Fish from both groups were then put back into a normal group setting. These early rearing conditions affected several aspects of social competence in a long‐term manner (published previously in Arnold and Taborsky 2010; Taborsky et al. 2012). Using quantitative real‐time PCR, we showed that the expression level of the candidate genes involved in the stress response (glucocorticoid receptors, corticotropin releasing factor) was permanently altered in the brain of these fish when they reached the adult stage (Taborsky et al. 2013). Our results suggest that the early rearing environment can thus induce permanent reprogramming that may directly affect social behaviour in a fish, as found in mammals. Uncovering which molecular mechanism (gene expression, epigenetic marking) is altered in association with permanent deleterious changes in social behaviour allows us to start understanding how such a complex social behaviour is normally built (Soares et al. 2010). The fact that some genes involved in the stress axis were altered in their expression by the early rearing environment in the same way as found in mammals gives us a handle on the evolution of behaviour at the mechanistic level. It sheds light on a potential molecular convergence that could underly the observed convergence of the behavioural consequences of the early social environment.

### 3‐Natural variation in threespine sticklebacks

Understanding the evolution of behaviour necessitates understanding the natural variation available for selection to act on. However, the natural variation in its underlying mechanistic bases is quantified simultaneously in surprisingly few cases. When this has been carried out in wild individuals, large interindividual variation in molecular, cellular and physiological levels were found in nature (see Williams 2008 for examples at the hormonal level). During my postdoctoral work, we studied variation in gene expression among individuals of the same phenotype and found that we could classify individuals to their phenotype using only their brain gene expression profiles [dominant/subordinate (Renn et al. 2008), sneaker/immature (Aubin‐Horth et al. 2005a), male/female (Renn et al. 2008)], but not in all cases (cooperative breeding species, Aubin‐Horth et al. 2007), which was intriguing. Furthermore, some of the genes that were highly differentially expressed between dominant and subordinate males in *A burtoni* were the ones that varied the most among individuals of the same dominance status (Renn et al. 2008).

In 2009, my laboratory moved to the Institut de Biologie Intégrative et des Systèmes (IBIS) at Université Laval, where I am now an associate professor in the Biology Department. My laboratory wanted a model in which to study genetic and plastic variation in behaviour and its associated molecular variation. We chose the threespine stickleback (*Gasterosteus aculeatus*). It is small (5–10 cm), it has a short life cycle (matures in 1 year) and can be bred in the laboratory. It is found all over the northern hemisphere, in saltwater & freshwater. It invaded freshwater in a replicated fashion from a refuge saltwater population after the last glaciation that stood over the northern hemisphere, making it an excellent species to study evolution in new environments. Moreover, its behaviour has been studied for 50 years (mostly in the freshwater form), its hormonal system in relationship to reproductive behaviour is also well studied, and it has a sequenced genome. We started using this model fish species to answer our questions about the mechanisms underlying natural variation in behaviour. We focused on five behaviours: boldness, aggressiveness, activity in a familiar environment, exploration of a novel environment and sociability. These behaviours have been shown to vary among individuals, populations and species and have the potential to have a strong influence on fitness. Furthermore, these behaviours are studied in several vertebrates, allowing us to make parallels with studies in birds and mammals. (Réale et al. 2007).

#### Interindividual variation in behaviour and mechanisms

Among individual variation in behaviour within a population is common and usually forms a continuum at the population level. The response expressed by an individual to a stimulus has been often investigated in the context of ‘personality’ or ‘coping style’ but is also studied without being put in this framework (Koolhaas et al. 1999; Réale et al. 2007). Several lines of evidence suggest that variation in behaviour and in the stress response are linked. For example, different coping styles in laboratory rodents have been associated with different stress reactivity (Koolhaas et al. 1999). Also, artificially selected lines of rainbow trout selected for a high or low hormonal response to stress also show divergence in behaviour even though it is not the target of selection (Øverli et al. 2005). Furthermore, knock‐outs and pharmacological manipulations of laboratory animals creating variation in molecular pathways implicated in the hormonal stress response have been shown to have effects on behaviour (Backström et al. 2011; Schjolden et al. 2009). In view of all these observations, we wanted to test whether there was an association between natural variation in behaviour in a population and variation in the stress response molecular pathway in threespine stickleback. We studied the expression of the candidate genes involved in the stress response molecular pathway in the brain using quantitative real‐time PCR. We found that boldness and aggressiveness towards a conspecific covaried in territorial and reproductive males. Furthermore, we showed that these behaviours were negatively associated with cortisol levels and positively with the expression levels in the brain of genes involved in the stress response (glucocorticoid receptors, Aubin‐Horth et al. 2012). These fish were wild caught as adults, such that the differences observed in behaviour and brain gene expression were potentially the result of genetic variation but also of the environment, including learning (see Box 2). Our study thus opened up a window on the natural variation in the stress response cascade, supporting the idea that we must study individual variation of hormonal systems to understand the evolution of behaviour (‘fight the tyranny of the golden mean’, Williams 2008). Our results also showed that there is a substrate for natural selection to act on. This work highlighted the fact that studies in controlled rearing environment that combine information on the molecular mechanism associated with and implicated in this behavioural variation would be advantageous.

Box 2 **Genetic variation and phenotypic plasticity**

Differences in behaviour between individuals, populations and species can result from genetic variation. Artificial selection lines provide us with a striking example of the potential for selection to act on behaviour variation. When behaviour has a high heritability, this selection pressure results in a measurable evolutionary response. For example, docility in wild fox (Trut et al. 2004) and in rats (Albert et al. 2008) has been selected to result in extremely docile and extremely aggressive individuals towards humans. Diverse behaviours that are expressed in different contexts have been shown to respond readily to selection, for example, propensity for wheel running in mice (Koteja et al. 1999), risk‐taking behaviour in a bird (Van Oers et al. 2004), predator avoidance in a gastropod (Dalesman et al. 2009) and maternal aggressive defence behaviour in mice (Gammie et al. 2006). Genetic variation has also been found for hormone levels and molecular networks, for example using selection lines targeting the stress response axis in rainbow trout (Overli et al. 2002) or studying variation in the thyroid hormone physiological regulatory network among wild populations of threespine stickleback (Kitano et al. 2010).

Behaviour variation is also found within the life of an individual and result from exogenous and endogenous environmental effects (phenotypic plasticity, West‐Eberhard 2003). Different categories of behavioural plasticities are found, including environmental effects during development (developmental plasticity, sometimes referred strictly to as ontogenetic plasticity), as the result of acclimation, through learning, and as short‐term responses to stimuli (called contextual or activational plasticity, see Snell‐Rood 2013 and Stamps 2015 for complete reviews). Developmental plasticity can give rise to a continuous distribution of behaviour (Denver et al. 1998). It can also result in distinct alternative behaviours, for example courting and noncourting males (Moczek and Nijhout 2003; Tomkins and Brown 2004). Acclimation to a situation is the process of adjusting to a change in the environment, usually at the physiological level. It can also be measured in behaviour, such as in a shift of habitat use or the use of air breathing in fish faced with hypoxia (Chapman and Mckenzie 2009). This plastic behaviour variation can happen in all life stages. A shift in behaviour can also be the consequence of learning (Snell‐Rood 2013). Finally, individuals spend most of their lives modulating their behaviour on a very short time scale in response to external and internal stimuli (Soares et al. 2010; O'Connell and Hofmann 2011). These different types of plasticity are also found for hormonal (Cuddy et al. 2012) and molecular networks (Landry et al. 2006).

There are also instances of genetic variation for the response to an environmental cue, with some individuals responding more than others. This is genetic variation for plasticity (West‐Eberhard 2003). If this genetic variation is found within a population, it means that selection for the plastic response an individual expresses could result in an evolutionary response. This genetic variation for plasticity could also be the result of past selection, for example when comparing the reproductive behaviour of two populations (Boersma et al. 1998; Foster 1999). Responses that could vary between genotypes include the propensity to join a group, to attack a conspecific, to flee a predator, to eat a novel food and to court a potential mate (Snell‐Rood 2013). If the response to a challenge gives higher fitness to an individual than another response, and there is genetic variation underlying this difference in response, then even a ‘plastic’ behaviour can evolve across generations (Dalesman et al. 2009). Genetic variation for plasticity is also found in endocrine and molecular networks. For example, it has been shown between populations for hormonal responses to a stressor (Dahl et al. 2012). It has also been shown using whole‐genome transcriptomic responses between genetically differentiated lines facing different levels of nutrition stress (Landry et al. 2006) and between populations facing different temperatures (Morris et al. 2014).

#### Behaviour variation between populations

An evolutionary response will result from natural selection if a large proportion of the phenotypic variation selection acts on is the result of additive genetic variance rather than of environmental effects (see Box 2). We studied the five behaviours mentioned above in juvenile threespine sticklebacks reared in a common environment, to minimize these environmental effects and detect genetic variation in behaviour. These individuals were the offspring of wild parents originating from two freshwater populations that differed in their antipredator morphology and probability of predation. We demonstrated that aggressiveness towards a conspecific, boldness towards a predator and activity in a familiar environment differed between populations, although the juveniles studied had never encountered a predator and the ecological conditions faced by their parents (Lacasse and Aubin‐Horth 2012). Juveniles whose parents showed an underdeveloped antipredator morphology (shorter dorsal and pelvic spine, smaller or absent pelvic girdles and pelvic spine) and lived in a lake with a lower predation probability were bolder, more active and more aggressive and vice versa for the population facing higher predation probability. Our observations thus supported the hypothesis of behaviour and morphology cospecialization, rather than trait compensation (DeWitt et al. 1999; Mikolajewski et al. 2010). The proximal hormonal and molecular mechanisms leading to this phenotypic integration between morphology and behaviour are still to be uncovered (Box 4). Using this model system to study mechanisms will allow us to test predictions that can be made for each potential cause of this phenotypic integration (natural selection, pleiotropy and plasticity, see Box 4).

We also studied the negative relationship between individual aggressiveness towards a conspecific and sociability (measured as tendency to swim with a group of conspecifics) in juveniles originating from these two populations and reared in a controlled environment (Lacasse and Aubin‐Horth 2014). Interestingly, highly aggressive individuals that were also highly social were absent in both populations, suggesting a conflict between competition (being aggressive) and cooperation (spending time in a group). A significant negative association between being aggressive and social was observed only in the juveniles whose parents originated from a population that faced a high predation probability. There was no significant correlation between these two behaviours in the population facing a low predation risk. This suggests that this association is not the result of a genetic constraint and thus has the potential to evolve and that there may be differential selection pressures acting in these two populations (Lacasse and Aubin‐Horth 2014).

#### Quantifying natural variation in behaviour and molecular networks at the same time

Comparing populations that have evolved separately from each other allows to test whether genetic divergence in behaviour is associated with physiological and molecular genetic divergence. It also allows determining at the molecular level what types of biological functions evolve between these populations that diverge in behaviour. This type of information sheds light on the origin of this behavioural divergence, including natural selection, genetic drift, pleiotropic effects and selection on divergent reaction norms (see Box 3 and 4). Threespine sticklebacks show high divergence in morphology and physiology between individuals of marine origin and individuals from freshwater populations (Bell and Foster 1994). Although there is a high potential for behaviour to also diverge in a new environment, we know much less about behaviour variation and its underlying mechanisms between marine and freshwater sticklebacks (Bakker and Feuth‐de Bruijn 1988; Messler et al. 2007; Wark et al. 2011). We studied common‐environment reared juveniles whose parents originated from a marine and a freshwater population of sticklebacks. We quantified the behaviour divergence associated with the colonization of the novel freshwater habitat after the last glacial retreat in North America (Di‐Poi et al. 2014). We found that freshwater sticklebacks were less social, more active and more aggressive than juveniles of marine origin (Di‐Poi et al. 2014). We concurrently studied the molecular and physiological correlates of this divergence in behaviour. We uncovered large genetic divergence between the two populations in stress reactivity (C. Di‐Poi, J. Lacasse, S. Rogers and N. Aubin‐Horth, unpublished manuscript) and in four physiological regulatory networks implicated in the stress response and in social behaviour (C. Di‐Poi, D. Bélanger, M. Amyot, S. Rogers and N. Aubin‐Horth, unpublished manuscript). All these studies show that genetic variation in behaviour is widespread in threespine stickleback, making it an excellent model to quantify natural variation in specific candidate molecules and in genomewide changes, for example using RNA‐seq and proteomics.

Box 3 **What I talk about when I talk about mechanisms**

Studying molecular changes is central to understanding the mechanisms behind behaviour diversity, which interact with the neuronal basis of behaviour. These changes include gene transcription (mRNA levels & epigenetic modifications that affect these mRNA levels: DNA methylation, histone post‐translational modifications and micro RNAs) and proteins (their quantity, activity, localization in the cell, interaction with other proteins and their post‐translational modifications). Furthermore, the physiology of an organism, which often results from the molecular changes listed above, is also associated with behavioural variation (Black et al. 2005; Dantzer et al. 2008; de Bekker et al. 2013). Behaviour can be modified in direct and indirect ways by all these mechanisms, and we now know that in turn behaviour can affect several of these processes. We have particularly detailed information on these two‐way interactions in endocrine systems. For example, in the loser effect, an individual that loses a fight shows changes in sex steroid levels, and this in turn affects its aggressiveness, which lower its probability of winning the next fight (Earley and Hsu 2008; Oliveira et al. 2009). The fact that these molecular changes result from responses to the environment and can happen very fast must be taken into account when designing an experiment and performing sampling. This is essential to take a snapshot of these mechanisms in the right timeframe.

Note that the genetic variation that leads to these behavioural differences is not included in this broad definition of mechanisms, as the candidates uncovered by genetic studies (point mutations, indels, inversions, etc. in coding and noncoding regions) and by studies of mechanisms do not always correspond. Indeed, one genetic modification may affect several downstream processes, which are reflected at different times in the life of the organism and at different levels of biological complexity. Therefore, studies that uncover genetic bases and molecular changes complement each other and should not be expected to always reveal the same players (Bell and Aubin‐Horth 2010).

Box 4 **Integrative biology**

Behaviour does not evolve in isolation from the other traits of an organism, making it essential to connect information on several levels of biological complexity (molecular, cellular, physiological, morphological, behavioural and life‐history traits). The integration of phenotypes can be quantified as the correlation between traits of interest (Pigliucci 2003). The degree of integration of these traits (the strength of correlation) is the result of the interaction of (i) the effects of selection, (ii) of constraints (phylogenetic, genetic and developmental, see Pigliucci 2003) and (iii) the level of plasticity of these traits in response to environmental conditions (Schlichting 1989). For example, an association between a morphological and a behavioural trait may be present in one population and not in another. This difference in integration could have different reasons. It could be because individuals in the two populations face different selection pressures that affect the tightness of this relationship if only one trait is under selection or if the correlation between the two traits increases fitness only in certain conditions. It could be because the product of a single gene affects all these traits, called structural pleiotropy [to distinguish it from the general term ‘pleiotropy’ that is often used in quantitative genetics (Van Oers et al. 2005)]. Finally, it could be because they have not faced the same environment within their lifetime, resulting in plastic changes in one or many traits that affect the correlation (Bell and Sih 2007). Distinguishing between these hypotheses necessitates knowing the underlying mechanism resulting in this behavioural and morphological variation.

Comparative physiologists have pioneered this type of integrative work. For example, female crickets of the *Gryllus* genus can develop into two forms with distinct dispersal behaviour (called a polymorphism): a flightless form with short wings, very low investment in flight muscle growth but large gonads, and a flying form, with long wings, large flight muscles but smaller gonads (Zera and Huang 1999). Behaviour, physiology, morphology and life‐history traits are integrated in two distinct forms. Studying differences in hormonal systems during the development of these two female forms uncovered a significant difference in the activity of the juvenile‐hormone esterase enzyme, which controls the degradation of juvenile hormone. This hormone is functionally implicated in development in insects (Zera and Huang 1999). This integrated dispersal behaviour phenotype, resulting from changes in wing morphology and a modification in allocation of resources between somatic and gonadal growth, is thus associated with a large shift in the main hormonal axis controlling development of immature stages in crickets and insects in general. Whether this integrated phenotype is the result of selection, pleiotropy or plasticity (as outlined above), or the result of more than one of these factors, could be tested in that model system. The study of mechanisms and resultant behaviour variations are clearly important to study together.

## Conclusion

A fruitful new avenue in the study of the mechanisms underlying behaviour variation is the ecological annotation of genes. As more and more information on the underlying mechanisms of variation in the same behaviours in different species emerges, we will be able to pair a gene to a function in a given species and context (Landry and Aubin‐Horth 2007; Aubin‐Horth and Renn 2009; Pavey et al. 2012). The first step we can take towards creating such an ecological annotation of genes is to associate phenotypes with changes in molecular, cellular and physiological traits (defined as a ‘causal role’ by Doolittle et al. 2014; Aubin‐Horth and Renn 2009; Pavey et al. 2012). In the longer run and in species in which this is feasible, this ecological annotation will be completed using functional analyses, as functions may be species‐specific and some genes may only be found in certain species (Matzkin 2014). The association of an organism feature with a molecular phenotype in different species or contexts can be interesting to start uncovering the function of genes resulting from a duplication (termed paralogs), as seen in the fish‐specific whole‐genome duplication (Meyer and Van de Peer 2005; Machado et al. 2014). These fish‐specific paralogs are found as a single copy in common model systems such as humans. However, it is challenging to assign a function to paralogs in a nontraditional model system such as fish, as duplication has been shown to potentially result in the subdivision of the function of a gene between the two new copies (subfunctionalization) or the evolution of a novel function (neo functionalization) (Lynch and Force 2000). Creating these ecological annotations will thus certainly bring up numerous challenges as well as new insights. Also, while the focus has been put on variation in mRNA levels in the last years, more and more studies provide information on other biological levels that may be implicated in behaviour variation (see Box 3 ‘what I talk about when I talk about mechanisms’ for examples). Among these, epigenetic modifications that affect mRNA levels such as DNA methylation and microRNAs and that have been directly implicated in behaviour variation are an already fruitful focus (DNA methylation in honeybee, Kucharski et al. 2008; microRNA in mice, Tan et al. 2013).

Tinbergen noted in 1963 in the conclusion of his paper presenting the categories of explanations of behaviour, now known as ‘Tinbergen's four questions’: ‘*What I have been at pains to develop is the thesis that we are witnessing the fusing of many sciences, all concerned with one or another aspect of behaviour, into one coherent science, for which the only correct name is “Biology of behaviour”*’. Fifty years later, we are harvesting the fruits originating from this encouragement to address questions about behaviour variation from the complimentary angles of evolution and mechanisms. We are far from having answered all these interesting questions, but we are well equipped to do so.
